# Virtual Reality for Preoperative Anxiety in Patients Undergoing Odontectomy Under General Anesthesia: Protocol for a Randomized Controlled Trial

**DOI:** 10.2196/94535

**Published:** 2026-05-26

**Authors:** Saptiadi Oktora, Harmas Yazid Yusuf, Eriska Riyanti, Firdaus Hariri

**Affiliations:** 1 Doctoral Programme, Faculty of Dentistry, Universitas Padjadjaran, Jalan Sekeloa Selatan No.1, Bandung, West Java, 40132, Indonesia, 62 81320192185; 2 Oral and Maxillofacial Surgery Department, Faculty of Dentistry, Universitas Padjadjaran, Bandung, West Java, Indonesia; 3 Pediatric Dentistry Department, Faculty of Dentistry, Universitas Padjadjaran, Bandung, West Java, Indonesia; 4 Oral and Maxillofacial Surgery, Faculty of Dentistry, Universiti Malaya, Kuala Lumpur, Wilayah Persekutuan, Malaysia

**Keywords:** odontectomy, preoperative anxiety, virtual reality, general anesthesia, salivary alpha-amylase, salivary cortisol, randomized controlled trial

## Abstract

**Background:**

Odontectomy is a common surgical procedure often associated with significant preoperative anxiety, particularly when performed under general anesthesia. Anxiety can lead to physiological changes, such as increased blood pressure and heart rate, and may affect anesthetic requirements and postoperative recovery. Virtual reality (VR) has emerged as a promising non-pharmacological intervention to reduce anxiety through immersive distraction.

**Objective:**

This study aims to evaluate the effectiveness of VR in reducing preoperative anxiety in patients undergoing odontectomy under general anesthesia. The study will assess anxiety using both subjective measures (Amsterdam Preoperative Anxiety and Information Scale, APAIS) and objective biomarkers (salivary alpha-amylase, salivary cortisol, and vital signs).

**Methods:**

This is a prospective, randomized controlled trial. Patients aged 17‐40 years undergoing odontectomy under general anesthesia at the Dental and Oral Hospital, Universitas Padjadjaran, will be recruited. Participants (n=32) will be randomly assigned to either the intervention group (VR experience of the operating room) or the control group (standard verbal education). Data collection includes salivary samples (alpha-amylase and cortisol), vital signs (blood pressure, heart rate, respiratory rate), and APAIS scores measured at baseline, immediately post-intervention, and 15 minutes post-intervention. Data will be analyzed using independent and paired *t* tests or Mann-Whitney and Wilcoxon tests, depending on normality.

**Results:**

The study is scheduled to be conducted between October 2026 and December 2026. The findings will provide evidence regarding the efficacy of VR as a standard anxiolytic tool in oral surgery.

**Conclusions:**

If effective, VR could serve as a non-invasive, safe alternative to pharmacological premedication for managing preoperative anxiety, potentially improving surgical outcomes and patient comfort.

## Introduction

Odontectomy, the surgical removal of impacted third molars, is one of the most frequent procedures in oral and maxillofacial surgery [1,2]. Due to the complexity of the procedure and the use of general anesthesia, patients often experience high levels of preoperative anxiety [3]. This anxiety is a physiological and psychological response to the anticipation of surgery, fear of pain, and loss of control associated with anesthesia [3].

High levels of preoperative anxiety trigger the hypothalamic-pituitary-adrenal (HPA) axis and the sympathetic nervous system, leading to the release of stress hormones such as cortisol and catecholamines [4]. Clinically, this manifests as elevated blood pressure, tachycardia, and tachypnea [5]. These physiological changes can complicate the induction of anesthesia, increase the requirement for anesthetic drugs, and negatively impact wound healing and immune function postsurgery [6,7].

Current management of preoperative anxiety often relies on pharmacological interventions, such as benzodiazepines, which may have side effects like drowsiness or respiratory depression [8]. Consequently, there is growing interest in nonpharmacological alternatives. Virtual reality (VR) is an immersive technology using head-mounted displays, such as the Oculus system, to provide a visual and auditory experience that distracts the patient and reduces anxiety by redirecting attention away from the surgical environment [9]. Over the last decade, numerous studies have evaluated immersive VR as a tool for reducing anxiety, pain perception, and distress in various medical and surgical procedures [9,10]. However, within the field of oral surgery—specifically for complex procedures under general anesthesia—there is a need for comprehensive research that combines subjective psychometric scales with objective biochemical stress markers [10]. Most existing dental VR literature focuses on pediatric populations or minor procedures, leaving a gap in evidence regarding its efficacy for adult patients undergoing oral surgery under general anesthesia [10].

This study aims to determine the effectiveness of VR in reducing preoperative anxiety by analyzing changes in the Amsterdam Preoperative Anxiety and Information Scale (APAIS) scores, vital signs, and specific stress biomarkers—salivary alpha-amylase (sAA) and salivary cortisol [10,11].

## Methods

### Study Design and Setting

This study uses a quantitative experimental design with a randomized pretest and posttest control group structure. The research will be conducted at the Dental and Oral Hospital (RSGM) of Universitas Padjadjaran, Bandung, Indonesia. The protocol is designed in accordance with the Consolidated Standards of Reporting Trials (CONSORT) guidelines for nonpharmacological interventions.

### Participants

Participants will be recruited using purposive sampling based on specific eligibility criteria. Inclusion criteria comprise patients aged 17‐40 years who are scheduled for odontectomy of at least two impacted third molars under general anesthesia. Eligible patients must be in good systemic health (American Society of Anesthesiologists, ASA physical status I or II) and have no prior history of surgery involving general anesthesia. To control for confounding factors affecting stress biomarkers, patients will be excluded if they have a history of endocrine disorders, chronic stress conditions, sleep disorders, or are currently taking medications that alter the HPA axis or autonomic response, such as anxiolytics, antidepressants, corticosteroids, or beta-blockers. Patients with visual or hearing impairments that prevent the use of VR will also be excluded. While patients with severe diagnosed psychiatric disorders are excluded to minimize profound psychopathological confounding, patients with general dental anxiety or phobia remain eligible to ensure the clinical generalizability of the findings.

### Randomization and Blinding

Eligible participants will be randomly allocated (1:1 ratio) into either the Intervention (VR) or Control group. To ensure proper allocation concealment, a computer-generated randomization sequence will be created by an independent researcher not involved in clinical care or data collection. The allocation sequence will be concealed using sequentially numbered, opaque, sealed envelopes, which will only be opened after the patient has consented to participate. Due to the nature of the VR intervention, blinding of patients and the administering clinician is not possible. However, the study will use an assessor-blinded approach; laboratory personnel analyzing the salivary biomarkers, and statisticians conducting the data analysis will be strictly blinded to group assignments.

### Intervention

To ensure a standardized immersive experience and minimize confounding variables, all study procedures—including the intervention and saliva collection—are scheduled to occur between 08:00 AM and 10:00 AM on the day before surgery, specifically to control for the circadian rhythm of salivary cortisol. Participants are randomly assigned to either the intervention or the control group. In the Intervention group, participants use a head-mounted display (Oculus Quest 2) to view a 10-minute 360-degree immersive video. This content, which has been previously piloted to ensure its clarity and tolerability, systematically simulates the patient’s journey from entering the operating room to the induction of anesthesia, aiming to provide psychological desensitization to the surgical environment. In contrast, the Control group receives 10 minutes of standard verbal education regarding the surgical procedure. This session is supported by a flip chart featuring standard images of the preparation room, operating theater, and recovery room, ensuring the information provided is consistent with the visual content of the VR intervention while lacking the immersive component.

### Outcomes and Data Collection

Data collection occurs at three standardized time points: Baseline (immediately before the VR or verbal education intervention), Post-test 1 (immediately after the intervention), and Post-test 2 (15 minutes after the intervention). The subjective anxiety assessment uses the APAIS, comprising 6 items scored on a 5-point Likert scale, with total scores ranging from 6 to 30, categorized from “Not Anxious” to “Panic.” Objective physiological assessments include blood pressure (systolic/diastolic), heart rate, and respiratory rate, which will be measured after the patient has rested for at least 5‐10 minutes.

For objective biochemical assessments, unstimulated saliva samples (4‐5 mL) will be collected via the spitting method at the three designated time points. To ensure optimal long-term preservation of the stress biomarkers prior to enzyme-linked immunosorbent assay (ELISA) analysis, samples will be initially frozen at −10 °C and subsequently transferred to a −80 °C freezer. Salivary Alpha-Amylase (sAA) will be analyzed to measure sympathetic nervous system activity, while Salivary Cortisol will be measured to assess HPA axis activity.

### Sample Size

The sample size was calculated using the formula for unpaired analytical studies with numerical data. Based on a 95% confidence level (Zα=1.96) and 80% power (Zβ=0.84), the minimum sample size is 16 participants per group, totaling 32 participants.

### Statistical Analysis

Data will be analyzed using SPSS version 25.0 (IBM Corp). Descriptive statistics will be used to summarize the demographic and clinical characteristics of the participants. The Shapiro-Wilk test will be utilized to assess the normality of the data distribution, given the sample size is less than 50. Because this study involves repeated measurements across three time points, repeated-measures Analysis of Variance (ANOVA) will be conducted for normally distributed data to evaluate the main effects of time, group assignment, and the time-by-group interaction. If the data violate the assumption of normality, non-parametric alternatives such as Generalized Estimating Equations (GEE) or the Friedman test will be applied. To comprehensively report the magnitude of the intervention’s effect, effect sizes (eg, partial eta-squared or Cohen *d*) and 95% confidence intervals will be presented alongside the *P* values. A *P* value of <.05 is considered statistically significant..

### Ethical Considerations

This study has been approved by the Research Ethics Committee of Universitas Padjadjaran (approval #344/UN6.KEP/EC/2026). Written informed consent will be obtained from all participants prior to enrollment, in strict adherence to the principles outlined in the Declaration of Helsinki.

## Results

The study is currently in the preparation phase. Data collection is scheduled to take place from May 2026 to July 2026. The analysis will focus on the differences in APAIS scores, sAA levels, cortisol levels, and vital signs between the two groups.

## Discussion

### Principal Findings

This protocol outlines a randomized controlled trial designed to evaluate the efficacy of immersive Virtual reality (VR) as a nonpharmacological anxiolytic intervention for adult patients undergoing odontectomy under general anesthesia. We hypothesize that the preoperative VR intervention will significantly mitigate anxiety compared to standard verbal education [10]. Specifically, we anticipate that patients exposed to the 360 degree virtual operating room environment will exhibit lower subjective anxiety scores on the APAIS, more stable physiological parameters (blood pressure, heart rate, and respiratory rate), and significantly reduced levels of stress-related salivary biomarkers, namely cortisol and alpha-amylase [10,11].

### Comparison to Prior Work

Previous research has consistently demonstrated the use of VR in reducing anxiety during various medical and dental procedures [9,10]. The underlying mechanism is largely attributed to immersive distraction by heavily engaging the visual and auditory cortices. VR limits the brain’s cognitive capacity to process distressing environmental stimuli and nociceptive signals [9]. Consequently, this sensory gating dampens the activation of the sympathetic nervous system and the HPA axis, which theoretically translates to stabilized vital signs (heart rate, blood pressure) and reduced secretion of stress-related biomarkers such as salivary cortisol and alpha-amylase [7,8]. Furthermore, effectively mitigating preoperative anxiety has profound downstream clinical implications. Existing literature suggests that lower preoperative stress strongly correlates with smoother intraoperative anesthetic induction, decreased anesthetic requirements, and a reduction in postoperative pain perception and analgesic consumption [5,6]. However, existing literature in oral and maxillofacial surgery has predominantly focused on pediatric populations or minor procedures performed under local anesthesia. By investigating adult patients undergoing complex odontectomy under general anesthesia and using a robust combination of subjective psychometric tools and objective biochemical markers, this study addresses a significant gap in the current literature.

### Implications and Future Directions

The clinical implications of this trial are substantial. VR was specifically selected as the intervention modality because of its unique capacity to induce a profound sense of “presence,” effectively isolating the patient from the anxiety-inducing sights and sounds of the preoperative environment [10]. Compared to traditional nonpharmacological modalities such as audio therapy, 2D informational videos, or standard verbal pamphlets, immersive VR provides a significantly higher degree of sensory gating, making the psychological distraction far more potent [9,10]. Furthermore, when compared to standard pharmacological anxiolysis (eg, benzodiazepines), VR offers a safe, noninvasive, and easily reproducible alternative. It completely circumvents the inherent risks associated with sedative medications, such as respiratory depression, paradoxical reactions, and prolonged postoperative recovery times, factors that often contribute to patients’ underlying fear of general anesthesia itself [4]. If the hypothesized outcomes are achieved, immersive VR could fundamentally shift the paradigm of preoperative care in oral and maxillofacial surgery. This shift would not only enhance patient safety but also optimize hospital workflow by streamlining anesthetic induction and accelerating patient discharge. Future research should build upon these findings by evaluating the long-term impact of preoperative VR on postoperative variables, such as pain perception trajectories and wound healing, or by comparing different VR content to determine the most effective desensitization protocols.

### Strengths and Limitations

A key strength of this study is the multimodal approach to measuring anxiety, utilizing non-invasive salivary biomarkers alongside validated psychometric scales. However, several limitations must be acknowledged. First, the relatively small sample size and the single-center design may restrict the generalizability of the findings to broader populations. Second, the nature of the VR intervention inherently precludes blinding of the patients and administering clinicians. Furthermore, the novelty of the VR technology could induce placebo or expectancy effects, where the patient’s anticipation of receiving a “high-tech” intervention alters their anxiety response independently of the actual VR content. Future multi-center trials with larger cohorts are recommended to validate these findings.

The authors thank the staff at the Dental and Oral Hospital Universitas Padjadjaran and the Clinical Pathology Laboratory of Dr. Hasan Sadikin Hospital for their support.

## Abbreviations

APAIS: Amsterdam Preoperative Anxiety and Information Scale
ASA: American Society of Anesthesiologists
CONSORT: Consolidated Standards of Reporting Trials
ELISA: enzyme-linked immunosorbent assay
HPA: hypothalamic-pituitary-adrenal
sAA: salivary alpha-amylase
VR: virtual reality
